# Evaluation of a Prehospital Rotation by Senior Residents: A Web-Based Survey

**DOI:** 10.3390/healthcare9010024

**Published:** 2020-12-29

**Authors:** Laurent Suppan, Michèle Chan, Birgit Gartner, Simon Regard, Mathieu Campana, Ghislaine Chatellard, Philippe Cottet, Robert Larribau, François Pierre Sarasin, Marc Niquille

**Affiliations:** 1 Division of Emergency Medicine, Department of Anaesthesiology, Clinical Pharmacology, Intensive Care and Emergency Medicine, Faculty of Medicine University of Geneva, Geneva University Hospitals, CH-1211 Geneva, Switzerland; michele.chan@hcuge.ch (M.C.); birgit.gartner@hcuge.ch (B.G.); simon.regard@hcuge.ch (S.R.); mathieu.campana@hcuge.ch (M.C.); ghislaine.chatellard@hcuge.ch (G.C.); pinfico@gmail.com (P.C.); robert.larribau@hcuge.ch (R.L.); francois.sarasin@hcuge.ch (F.P.S.); marc.niquille@hcuge.ch (M.N.); 2 Division of Anaesthesiology, Department of Anaesthesiology, Clinical Pharmacology, Intensive Care and Emergency Medicine, Faculty of Medicine University of Geneva, Geneva University Hospitals, CH-1211 Geneva, Switzerland

**Keywords:** postgraduate medical education, prehospital, emergency medicine, leadership, non-technical skills

## Abstract

The added value of prehospital emergency medicine is usually assessed by measuring patient-centered outcomes. Prehospital rotations might however also help senior residents acquire specific skills and knowledge. To assess the perceived added value of the prehospital rotation in comparison with other rotations, we analyzed web-based questionnaires sent between September 2011 and August 2020 to senior residents who had just completed a prehospital rotation. The primary outcome was the perceived benefit of the prehospital rotation in comparison with other rotations regarding technical and non-technical skills. Secondary outcomes included resident satisfaction regarding the prehospital rotation and regarding supervision. A pre-specified subgroup analysis was performed to search for differences according to the participants’ service of origin (anesthesiology, emergency medicine, or internal medicine). The completion rate was of 71.5% (113/158), and 91 surveys were analyzed. Most senior residents found the prehospital rotation either more beneficial or much more beneficial than other rotations regarding the acquisition of technical and non-technical skills. Anesthesiology residents reported less benefits than other residents regarding pharmacological knowledge acquisition and confidence as to their ability to manage emergency situations. Simulation studies should now be carried out to confirm these findings.

## 1. Introduction

Prehospital emergency medicine has only been implemented in some emergency medical systems. The training and experience of the prehospital physicians working in such systems vary considerably from one region to another [1]. In certain situations, such as cardiac arrest or major trauma, the presence of a physician in the field has been shown to improve the patients’ outcomes [2,3,4,5]. Study results are however inconsistent, probably owing to differences in training and exposition of the pre-hospital staff [6,7]. Indeed, lack of resources has led some prehospital emergency services to hire junior residents who have not yet acquired all the necessary skills and knowledge required to manage critical situations [8,9]. In such settings, suboptimal care may ensue [10,11]. Moreover, lacking the proper skills and knowledge required to take care of critical patients might result in significant psychological harm to these junior physicians [12,13,14].

Prehospital interventions might be beneficial to senior residents [15,16]. Indeed, working in the prehospital environment might help develop both technical and non-technical skills such as leadership and decision making [17,18]. To allow non-specialist physicians to work in this environment whilst maintaining a high standard of care, some systems have developed supervision mechanisms [19]. Senior residents working in a system allowing for both distance and direct supervision by senior specialist physicians might therefore benefit from a prehospital rotation.

In Switzerland, medical students must complete a six-year curriculum before graduating and becoming residents. Depending on the region, these junior physicians are sometimes called interns or assistants. Senior residents are physicians who are still in training, are not yet certified as specialists, and who have at least three years of postgraduate clinical experience. Although most senior residents will eventually become certified specialists, only some of them progress to the next rank and become registrars or fellows. Registrars are either already certified as specialists or in the process of being certified. Only a few of these registrars will be selected to become consultants or attendings. For the sake of clarity, we will only use the terms resident, registrar, and consultant throughout this manuscript.

Emergency medicine is not a board-certified medical specialty in Switzerland [20]. Rather, physicians willing to work in an emergency department or in the prehospital setting must first become specialists in another domain such as general internal medicine or anesthesiology. To be certified by the Swiss Society of Emergency and Rescue Medicine (SSERM), physicians must complete a subspecialty training after obtaining their main certification.

Regardless of the specialty they have chosen, residents must complete several rotations designed to help them acquire medical knowledge directly or sometimes distantly related to their future work. Most rotations last between three and six months. Although some rotations, such as the intensive care rotation, are shared by most residents regardless of their chosen specialty, some are highly specific. Anesthesiology residents are for example required to complete rotations in specific anesthesiology domains such as neuroanesthesiology, while internal medicine residents are offered rotations in medical subspecialties, such as cardiology.

In Geneva, the prehospital medicalization system is two-tiered: the first is called frontline while the second is called supervision. The frontline tier is manned by paramedics working alongside physicians, most of whom are senior residents in internal medicine, anesthesiology, or in the emergency department (ED). For these physicians, the SMUR (Service Mobile d’Urgence et de Réanimation—Mobile Emergency and Resuscitation Unit) rotation usually lasts between 3 and 6 months. Higher-ranking physicians, such as anesthesiology and ED registrars or even consultants, might also work shifts as frontline physicians. The supervisors who compose the second tier are all SSERM certified specialists and can be either registrars or consultants.

Frontline physicians usually complete their missions without being supervised. They can however ask for distance (phone) or direct supervision at any time, including at night and during weekends. In certain specific situations, such as major incidents or major traumas, the emergency medical call center (EMCC) directly dispatches the supervisor alongside the frontline physician. The proportion of supervised interventions varies depending on the nature of the intervention, and ranges from less than 1% for acute respiratory distress [21] to around 50% for cardiac arrests [19].

In Switzerland, advanced life support ambulances are usually staffed by two paramedics who autonomously handle most emergencies. These paramedics, who follow a three-year curriculum before graduating, work according to protocols designed by their medical director. In Geneva, the EMCC dispatches SMUR units along with ALS ambulances in less than 20% of cases [22]. Whenever a SMUR is dispatched, the prehospital physician is expected to take the lead of the mission and to make specific medical decisions according to the patient’s condition. Therefore, SMUR units have more diagnostic and therapeutic options than regular ALS ambulances, such as arterial blood gas analyzers, antiarrhythmics, and even thrombolytic agents. During a typical three-month rotation, frontline physicians complete more than 150 missions. All these physicians are therefore exposed to many different situations and gain significant prehospital experience in a relatively short amount of time.

Data showing that a prehospital rotation could be beneficial to senior residents is still scarce. Our goal was to analyze the questionnaires sent to senior residents who had just completed their prehospital rotation to assess the perceived added value of this rotation in comparison with other rotations. Our hypothesis was that senior residents would experience a definite improvement in their technical and non-technical skills after a supervised prehospital rotation.

## 2. Materials and Methods 

### 2.1. Study Design and Setting

This is a descriptive web-based study following the CHERRIES guidelines [23] and carried out at the mobile emergency and resuscitation unit (Service Mobile d’Urgence et de Réanimation, SMUR) of the Geneva University Hospitals. It is based on a retrospective analysis of data prospectively collected between September 2011 and August 2020.

Although such studies do not fall under the Swiss Federal Human Research Act [24], a jurisdictional inquiry (Req 2020-00981) was submitted to the regional ethics committee which issued a declaration of no objection.

### 2.2. Study Data

During the week following the end of their prehospital rotation, frontline physicians received a single email inviting them to complete an end-of-rotation survey. These emails were identical except for the link they contained, which embedded a unique token. Participation in this survey was not mandatory and no reminder was sent.

Two different systems were used over the studied period:

- From 2011 to 2018, a system based on a Joomla 1.6 (progressively updated to version 2.5) platform (Open Source Matters, New York, NY, USA) was used. The questionnaire was administered using the Survey Force Deluxe 3 component (Joomplace, Minsk, Belarus). Unique links were generated by this component, before being manually embedded in e-mails along with a standard accompanying message.
- Starting 2019, a Joomla 3 platform was used, and e-mails were automatically generated by the AcyMailing 5 component (Acyba, Lyon, France). The unique links redirected to a questionnaire created under CommunitySurvey Pro 5 (CoreJoomla, Hyberabad, India).

All the questions from the first version of the platform were copied verbatim under the second platform. No change was made to the questions since inception. Three questions were however added over time. In 2018, participants were given the possibility to enter a free-text comment at the end of the survey. In 2019, two questions related to safety issues were introduced. 

The evolution of the system was made unavoidable by technological advances, by the need of applying critical security updates, and by the obsolescence of certain components. The anonymization philosophy remained nevertheless unchanged. The unique tokens generated by either system were used to avoid double entries and authorized the physicians to resume the survey if they had been interrupted. No data linking the tokens to individual e-mail addresses were stored in the database, thereby guaranteeing anonymization.

An information and consent form was loaded when potential participants clicked on the unique link embedded in their invitation e-mail. Information regarding anonymization procedures and data management were displayed on this form. Participants were told that clicking on the “start” button meant consenting to the terms and conditions of the survey. They were informed that the main purpose of the questionnaire was to help improve the prehospital rotation, but that all recorded data could also be used for research purposes.

The questionnaire was divided into three sections. The first was used to gather data regarding the physician’s profile. Age and gender were not collected because these data could theoretically identify the participant. Indeed, the number of physicians performing a prehospital rotation each year is rather limited. The second section contained 46 questions based on 6-point Likert scales and was designed to assess different aspects of the prehospital rotation. Most of these questions were adapted from the 2010 version of a questionnaire sent by the Swiss institute for medical education to all residents training in certified Swiss centers [25]. The 6-point Likert scales were therefore directly derived from the original questionnaire and were not modified in order to allow comparisons between rotations. The last section included seven questions based on 5-point Likert scales which were used to assess the specific added value of the prehospital rotation (Supplementary Table S1). The odd number was used to allow the respondents to score a neutral value if they did not see any added value to the prehospital rotation but still found it as beneficial as other rotations.

### 2.3. Inclusion and Exclusion Criteria

All questionnaires marked as completed by the computer system were included. This parameter was automatically applied when the respondent clicked on the “submit” button located on the last page of the survey. Being able to click this button meant that all the compulsory questions were adequately completed and recorded.

On one of the first pages of the questionnaire, the physicians were asked whether they had just completed their first prehospital rotation or if they had been returning for a subsequent one. In this latter case, the questionnaires were excluded. Questionnaires completed by registrars were also excluded from the analysis. All these questionnaires are still available in the original database.

### 2.4. Outcomes

The primary outcome was a composite of the five-point Likert scales, i.e., those evaluating the perceived added value of the prehospital rotation in comparison with other rotations.

Secondary outcomes were the individual results of each of the seven 5-point Likert scales. Although an analysis of the specific dimensions explored by the questions adapted from the 2010 version of the Swiss institute for medical education questionnaire was also planned, the copyright holders did not grant the right to use these questions for publication. Therefore, only five specific items assessed by means of original questions based on 6-point Likert scales were analyzed (Supplementary Table S1).

### 2.5. Data Curation, Data Availability and Statistical Analysis

Data were exported from a MySQL compatible database to comma-separated value files. These files were then imported and analyzed in STATA 16.1 (Stata Corp, College Station, USA). Our original data has been deposited on Mendeley Data [26].

Descriptive statistics were used to present the characteristics of the survey participants. All Likert scales were analyzed as continuous variables using non-parametric tests given the limited sample size. When appropriate, results are reported as median (Q1; Q3). Five-point Likert scales were coded to range from −2 (much less beneficial) to +2 (much more beneficial), with a value of 0 coded when the participant answered, “equally beneficial.” All these questions were equally weighted to compute the composite primary outcome, and Cronbach’s alpha was used to check for internal consistency.

A pre-specified subgroup analysis was performed using a Kruskal–Wallis H test to search for differences according to the participants’ service of origin (anesthesiology, emergency medicine, or internal medicine).

P values below 0.05 were considered statistically significant.

## 3. Results

**Figure 1:**
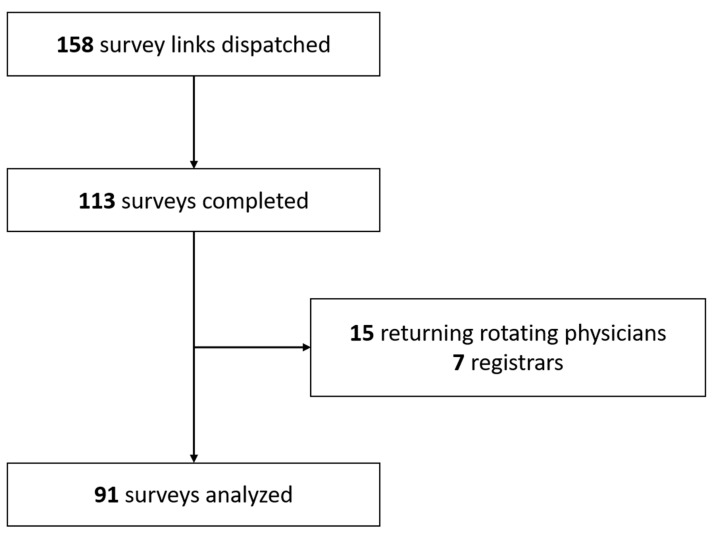
Experimental flowchart.

The completion rate was of 71.5% (113 surveys completed out of 158 invitations). After application of the exclusion criteria, 91 results were analyzed (Figure 1). The characteristics of the survey participants are detailed in Table 1.

**Figure 2:**
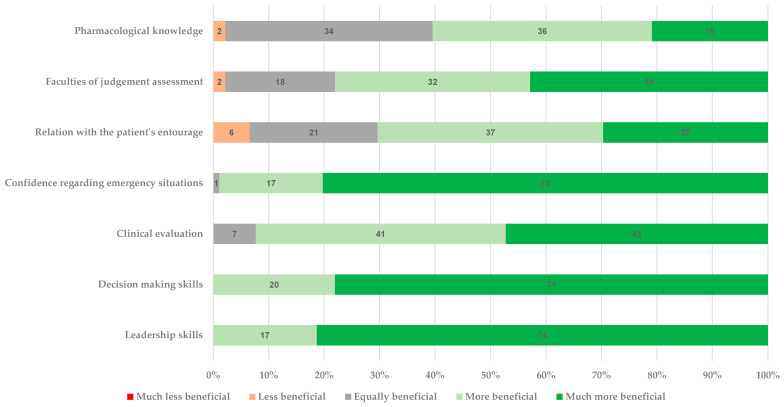
Added value of the prehospital rotation in comparison with other rotations.

Most senior residents found the prehospital rotation either more beneficial or much more beneficial than other rotations regarding the acquisition of technical and non-technical skills, with a median score of 1.3 (1.1;1.7) in the composite of seven questions. Detailed results are shown in Figure 2.

The improvement in leadership, decision making, and clinical evaluation skills was consistent regardless of the service of origin. This also held true for improvement in the ability to liaise with the patients’ entourage and to assess the patient’s faculties of judgement. While all residents reported a significant improvement in their confidence and ability to manage emergency situations thanks to the prehospital rotation, the effect was less important among anesthesiology residents (*p* < 0.001) (Supplementary Figures S2 and S3). The latter also described less improvement in their pharmacological knowledge (*p* < 0.001) compared to other residents. The internal consistency of the seven questions based on the 5-point Likert scale was adequate in this population of senior residents with an alpha value of 0.74.

The prehospital rotation was considered “excellent” by 75 participants (82%) and “very good” by the other 16 (18%). There was no difference according to the service of origin (*p* = 0.689). All residents answered that they would recommend a prehospital rotation to their peers, with 92% of them giving the highest rating on the 6-point scale. There was no significant difference between groups (*p* = 0.346).

The quality of teaching during prehospital interventions was highly rated by the senior residents, with a median of 6 (5;6) and no significant difference between groups (*p* = 0.10). Most participants also answered that supervisors could be reached in a timely manner, with 97% (110) of them giving the highest rating.

Individual comments recorded by the residents are reported in Supplementary Table S2 and available in the file containing the original study data [26].

## 4. Discussion

### 4.1. Main considerations

According to the results of our web-based questionnaire, senior residents report a subjective feeling that both their technical and non-technical skills have improved after a prehospital rotation. Regardless of their service of origin, all residents felt a significant improvement regarding leadership and decision making, two key non-technical skills. This is of particular importance as many of these senior residents will become registrars and will need to directly make use of these skills in their daily practice [27].

Two main reasons might help explain the improvement reported regarding non-technical skills. The first is linked to the in-hospital setting where senior residents spend the vast majority of their working hours. In this setting, residents seldom work alone and can always quickly call either a colleague or a registrar for help. Decisions are therefore often either shared with a colleague or taken by a higher-ranking physician. Given the relatively high number of healthcare workers involved in the management of hospitalized patients and the general lack of time constraint in this setting, in-hospital leadership is often less definite except in critical situations where it is commonly taken either by registrars or by consultants [28,29]. Conversely, critical situations are rather common in the prehospital setting and are most of the time managed by senior residents alone. Though supervisors can be called upon at any time, senior residents must continue taking care of their patients with the help of a limited number of paramedics while waiting for the specialist physician’s arrival. Clear leadership must therefore be swiftly and decisively established, and decisions must be taken in an unambiguous and timely manner [30,31]. The second reason for the reported improvement is related to the pathologies encountered during SMUR missions [32]. While adequate provision of advanced life support requires specific leadership and decision making skills, other missions require adapting the scope of these skills to particular situations. Among these, missions linked to psychiatric disorders [33], to suspected abuse or neglect [34], or to the assessment of the patient’s faculties of judgement are of special importance [35]. In the course of such missions, which amount to 10% of the SMUR’s activity, the prehospital physicians must often negotiate with patients and their relatives and, sometimes, with other partners (social workers, policemen, firefighters, general practitioners, etc.).

The prehospital rotation was felt to be less beneficial in certain areas depending on the residents’ service of origin. Senior residents coming from the anesthesiology department felt less improvement in their ability to manage emergency situations compared to their colleagues with an internal or emergency medicine background. Though this finding is easily explained as physicians working in the internal medicine department are less often confronted with emergency situations in their regular course of work, it is quite unexpected in emergency residents. Although measurement bias cannot be excluded, the most probable hypothesis is linked to a difference in supervision [28,29]. While senior anesthesiology residents often need to call on a supervisor in the case of an emergency but are usually alone in the operating room [36], immediate and direct supervision is always within reach in the emergency department. In this setting, senior emergency medicine residents might therefore never be called upon to manage critical emergencies on their own. Moreover, given their specific mindset, emergency medicine residents might be more interested in managing critical emergencies than anesthesiology residents and might therefore feel a more important improvement than their colleagues regarding this particular element.

The lesser improvement in pharmacological knowledge felt by senior anesthesiology residents compared to their colleagues is probably also linked to their specialty background. Indeed, while anesthesiologists often use inductor drugs such as etomidate or propofol, neuromuscular blocking agents such as suxamethonium or rocuronium, and powerful analgesics such as fentanyl or ketamine, most other residents have almost never used any of these drugs before their prehospital rotation [37].

The utility of sending physicians in the field to manage critical patients is still widely debated [38,39], even though physicians are increasingly involved in prehospital care [40]. While most studies focus on patient outcomes [5,41,42], only few have assessed how working in the prehospital setting affects physicians [8]. The results of our study shed a new light on the positive aspects prehospital rotations might have on senior residents. Further studies are of course needed to confirm these results and should include the use of simulations as well as objective assessments of technical and non-technical skills before and after prehospital rotations. Should such studies confirm our results, other medical training systems could choose to offer specialist-supervised prehospital rotations to senior residents.

### 4.2. Limitations

The main limitation of this study lies in its subjective nature. As this was only a web-based assessment, one can hardly be certain that senior residents will actually perform better when confronted with critical situations after having completed a prehospital rotation. Nevertheless, the answers given by these residents clearly show an increase in their confidence, which has been shown to be one of the key components required to efficiently manage emergency situations [43]. We were however unable to determine if and for how long this effect was sustained as we did not send follow-up questionnaires.

This study would have benefitted from a mixed-methodology. Unfortunately, free-text comments were only recorded since 2018, and the limited number of comments prevented us from carrying out a thematic analysis. Another limitation is that some interesting data had to be deleted from this manuscript as the copyright holders did not grant us the right to publish the results of most of the questions asked in the second tier of the online survey. While we failed to ask for the permission to reuse and adapt the questionnaire back in 2011, we cannot help but think that scientific data and methods should be widely accessible to allow for replication of previous findings and for improvement of previously designed methods. We support this philosophy by making our results freely available on Mendeley Data [26], by publishing our studies in open access journals, and by making our original questions free to reuse and easily accessible as supplementary material. We have recently gone even further by making original e-learning material freely available on the internet [44,45] to allow for replication of our findings or for testing hypotheses in different populations and urge our colleagues and fellow researchers to follow suit.

We must also acknowledge that both the overall rating and the probability of recommending a prehospital rotation might be overestimated as most of the senior residents performing a prehospital rotation have volunteered to do so. Moreover, as senior residents are asked to complete the questionnaire just after finishing their SMUR rotation, a “honeymoon” effect cannot be excluded. Finally, while a recall bias cannot be entirely excluded, the influence of such a bias on our results should be limited as all invitations were sent during the week following the end of the prehospital rotation and there were no reminders.

Although most senior residents reported an improvement in their leadership and decision making skills, their impression might not hold true in the field. Simulation studies assessing these non-technical skills both before and after a prehospital rotation should now be carried out to confirm these findings. Even though it was not specifically designed for this purpose, the NOTSS (Non-Technical Skills for Surgeons) framework could be used to assess non-technical skills [46]. This framework assesses five categories of non-technical skills which are as important to the prehospital physician as they are to the surgeon: leadership, communication and teamwork, situational awareness, decision making and task management.

## 5. Conclusions

This retrospective analysis of data prospectively collected over a period of 10 years shows that senior residents feel a significant improvement in their technical and non-technical skills following a prehospital rotation. While emergency medicine and internal medicine residents report a more significant benefit than anesthesiology residents regarding pharmacological knowledge and confidence in managing emergency situations, all senior residents report a greater improvement in these domains after a prehospital rotation than after other rotations.

Although the improvement reported in technical and non-technical skills are encouraging and could make stakeholders consider the creation of prehospital rotations (including in non-medicalized prehospital systems), their subjective nature is an undeniable limitation. Therefore, simulation studies should be undertaken to objectively assess the extent of the improvement regarding these skills, and, most importantly, leadership and decision making.

## Figures and Tables

**Table 1:** Characteristics of survey participants.

Characteristic		Anesthesiology Residents (*N* = 30)	Emergency Medicine Residents (*N* = 16)	Internal Medicine Residents (*N* = 45)
Years since graduation (median, Q1;Q3)		4 (4;6)	4 (3;5)	4 (4;5)
Target specialty ^1^				
	Anesthesiology (*n*, %)	30 (100%)	3 (19%)	2 (4%)
	Internal Medicine (*n*, %)	1 (3%)	11 (69%)	44 (98%)
	Other (*n*, %)	2 (7%)	5 (31%)	7 (16%)
Target emergency certification				
	Prehospital (*n*, %)	22 (73%)	7 (44%)	12 (27%)
	In-hospital (*n*, %)	3 (10%)	7 (44%)	11 (24%)

^1^ In Switzerland, residents can specialize in more than one domain.

## Data Availability

The data presented in this study are openly available in Mendeley Data at http://dx.doi.org/10.17632/hsrrjp88pc.2.

## Supplementary Materials

The following are available online at https://www.mdpi.com/2227-9032/9/1/24/s1, Table S1: Original questions and English translation, Table S2: Original comments made by rotating prehospital residents, Figure S1: Screen capture of the consent form displayed prior to starting the questionnaire, Figure S2: Confidence in emergency situations, by group, Figure S3: Pharmacological knowledge, by group.
