# Supplementary material for: Evaluation of a Prehospital Rotation by Senior Residents: A Web-Based Survey
Source: Healthcare (Basel). 2020 Dec 29;9(1):24. doi: 10.3390/healthcare9010024 (PMC7824315; doi:10.3390/healthcare9010024)

Supplementary Table 1

Original questions and English translation

Original question, in French	English translation
Likert scale : from -2 (beaucoup moins) to +2 (beaucoup plus). Par rapport à d'autres rotations, la rotation au SMUR vous a permis :	***Likert scale: from -2 (much less) to +2 (much more).*** Compared to other rotations, the prehospital rotation has allowed you:
De développer vos compétences d'évaluation clinique globale / votre « sens clinique »	To develop your clinical evaluation skills / your “gestalt”
De développer vos compétences relationnelles avec l’entourage des patients	To develop your social skills with the patients’ entourage
De développer vos capacités de « leader » d’une équipe	To develop your leadership skills
De développer votre capacité de prise de décision	To develop your decision making skills
De développer votre évaluation de la capacité de discernement des patients	To develop your assessment of the patients’ faculties of judgement
De développer vos connaissances pharmacologiques	To develop your pharmacological knowledge
De vous sentir plus à l’aise en cas d’urgence	To feel more confident regarding emergency situations
Likert scale : from 1 (inacceptable) to 6 (excellente)	***Likert scale: from 1 (unacceptable) to 6 (excellent)***
Au total, je trouve que la rotation au SMUR est :	Globally, I consider the prehospital rotation to be :
Likert scale: from 1 (ce n'est absolument pas exact) to 6 (c'est parfaitement exact)	***Likert scale : from 1 (this is absolutely not the case) to 6 (this is exactly the case)***
Recommanderiez-vous une rotation au SMUR?	Would you recommend a prehospital rotation?
Si j'ai besoin d'aide pour prendre une décision, je peux joindre un superviseur dans un délai raisonnable.	If I need help making a decision, I can reach a supervisor in a timely manner.
La qualité de l’enseignement lors des interventions est très bonne	The quality of teaching during prehospital missions is very good
La rotation au SMUR m’a permis d’acquérir une autonomie de fonctionnement	The prehospital rotation has enhanced my autonomy

Supplementary Table 2

Original comments made by rotating prehospital residents

Year	Service of origin	Comment
2020	Anesthésiologie	Je reviens quand?
2020	Médecine Interne	Arrivant à la fin de ma formation, l'expérience faite au sein de votre service était la meilleure que j'aie pu avoir. La politique de l'encadrement des internes et la culture de l'erreur permet une excellente sécurité des patients et la meilleure prise en charge qu'il puisse y avoir. Ma pratique au quotidien a vraiment été facilitée depuis le passage au SMUR.
2020	Anesthésiologie	Instaurer un journal club ou des colloque avec des articles/ littérature présente par les interne ou chefs de clinique serai à mon avis intéressant pendant ce tournus afin d'améliorer les connaissances
2019	Urgences	Merci! Gardez la même culture de l'enseignement.
2019	Médecine Interne	R.A.S. Super rotation.
2018	Médecine Interne	Mon passage au SMUR a été, de loin et pour tant de raisons, la meilleure rotation de ma formation post-graduée de médecine interne générale.
2018	Médecine Interne	Excellente rotation, sur tous les points. Formation de 4 jours au début de la rotation très appréciée. Bémol: protocoles doivent être mis à jour.
2018	Médecine Interne	J'apprécie énormément cette rotation, mon premier contact avec pré-hospitalier, je me suis sentie bien entourée et encadrée. A la fin de rotation je me suis sentie plus à l'aise dans certaines situations d'urgence, mais pour les développer plus je souhaiterai plus long durée de cette rotation pour s'exposer au maximum aux situations urgentes. Merci à toute l'équipe.
2018	Urgences	Tournus très apprécié. Notamment, le climat de confiance instauré par les cadres et les ambulanciers SMUR est propice à la critique constructive et à l'apprentissage.

Note: comments have been edited to correct spelling mistakes and typographical errors. The original comments can be found in the original data file uploaded on Mendeley Data (Suppan L, Niquille M – Prehospital Rotation Utility Dataset – Mendeley Data, 2020 – DOI: 10.17632/hsrrjp88pc.1).

Supplementary Figure 1 – Confidence in emergency situations

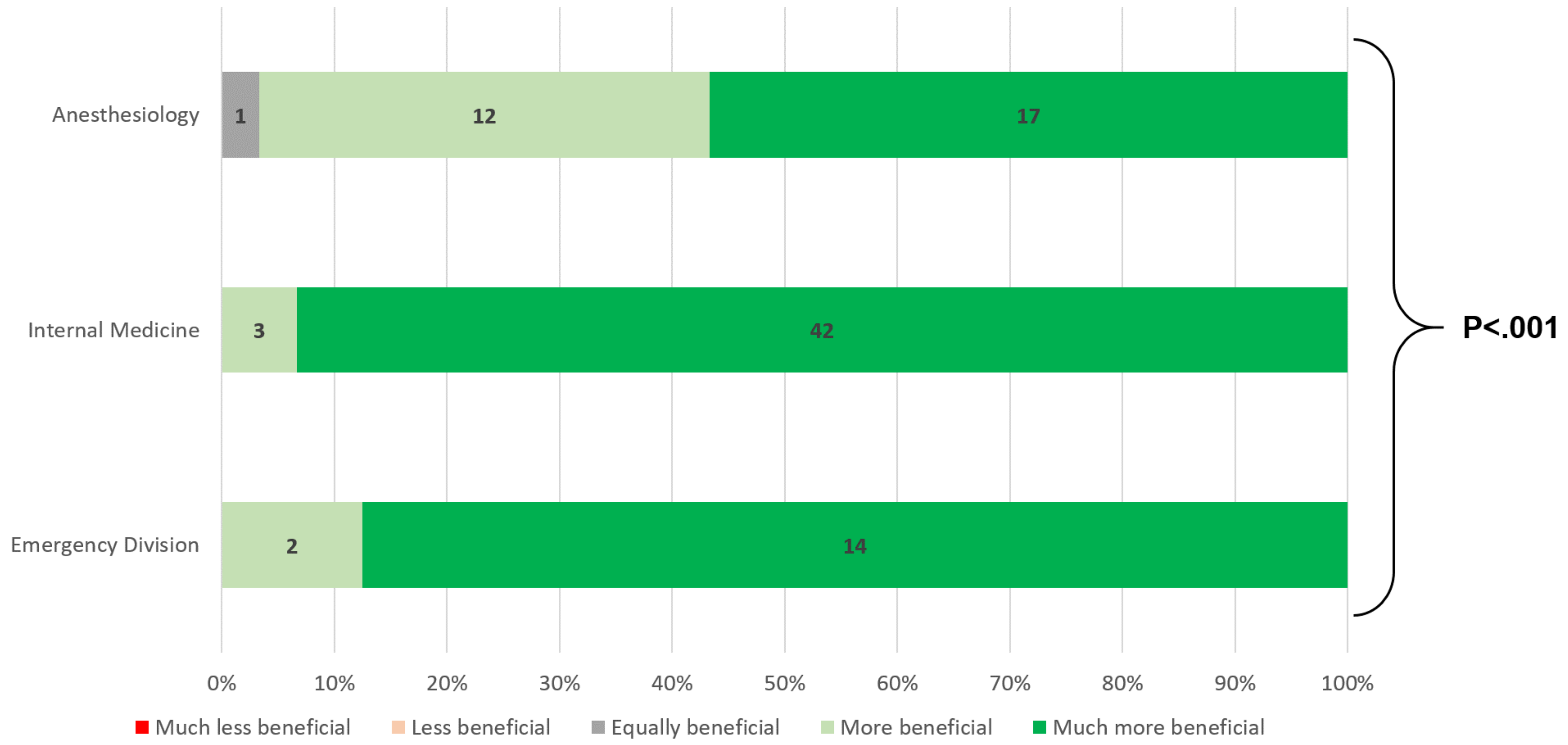

Supplementary Figure 2 – Pharmacological knowledge

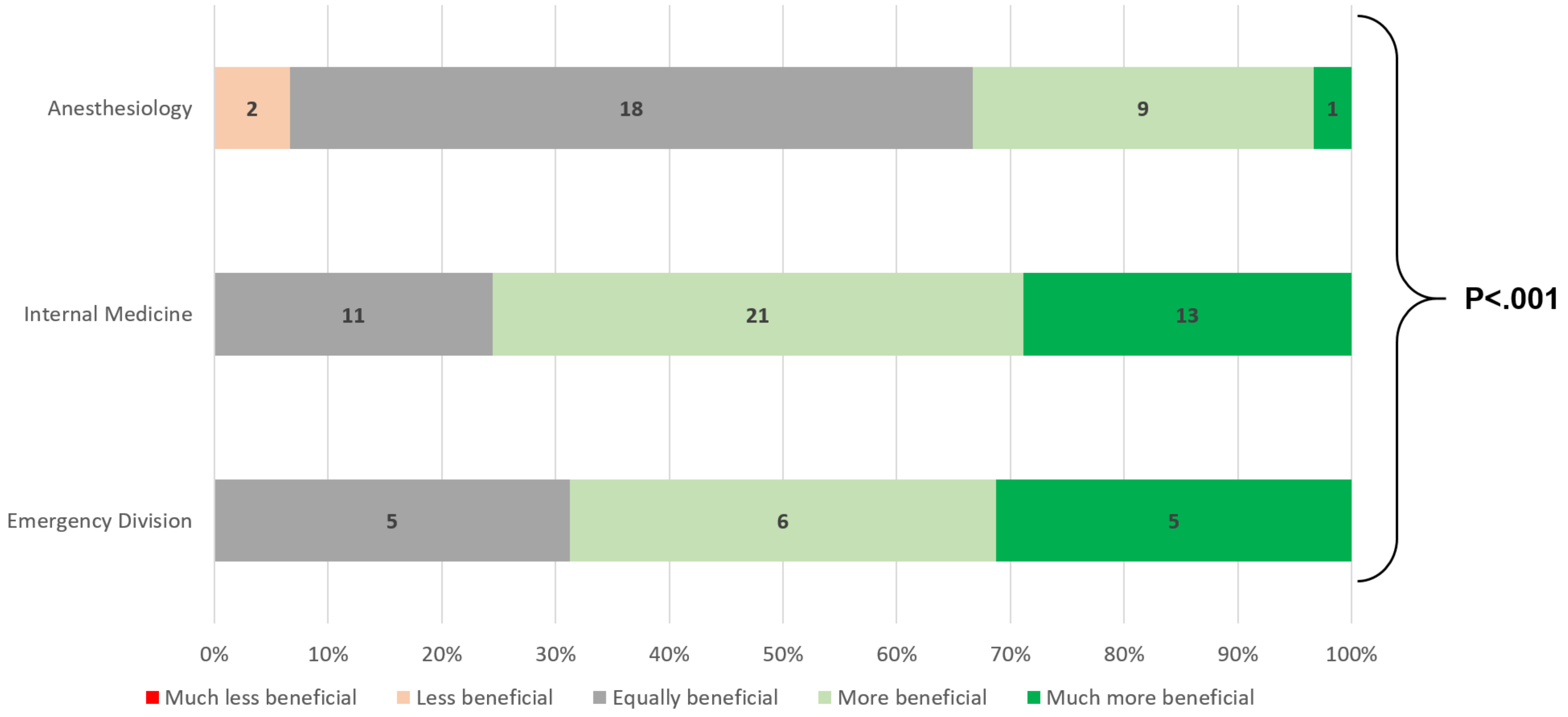

Supplement: Supplementary file 1 [file healthcare-09-00024-s001.pdf]
